# Short Communication: Evaluation of a New Rapid Diagnostic Test for Quality Assurance by Kala Azar Elimination Programme in Bangladesh

**DOI:** 10.1155/2011/862475

**Published:** 2011-11-02

**Authors:** Md Gulam Musawwir Khan, Mohammad Shafiul Alam, Abu Toha Bhuiyan, Maleka Arjumand Jamil, Bijoy Saha, Mazharul Islam, Rashidul Haque, Moazzem Hossain, Kazi M. Jamil

**Affiliations:** ^1^International Centre for Diarrhoeal Disease Research, Bangladesh (ICDDR,B), Mohakhali, Dhaka 1212, Bangladesh; ^2^Disease Control Unit, Directorate General of Health Services, Ministry of Health and Family Welfare, Government of Bangladesh, Mohakhali, Dhaka 1212, Bangladesh

## Abstract

In Bangladesh, serological tests have been widely used for the primary screening of visceral leishmaniasis (VL). Several serologic tests are available for the diagnosis of VL. Selection of the best test is important to permit diagnostic differentiation between symptomatic and asymptomatic patients and to reduce cross-reactivity. We evaluated the effectiveness of a new serological test “Onsite Leishmania Ab Rapid Test” as a part of “quality assurance” activities for the kala azar elimination programme of the Government of Bangladesh. Plasma samples of 100 parasitologically confirmed cases of VL along with 101 healthy controls were tested, and “Onsite Leishmania Ab Rapid Test” strip tests were positive in 94 out of 100 confirmed VL cases, whereas four out of 51 healthy subjects from the VL endemic areas also tested positive. All the 50 healthy volunteers tested negative. Thus, the sensitivity and specificity of “Onsite Leishmania Ab Rapid Test” strip test were found to be 94% (95% CI: 87–98) and 96% (95% CI: 90–99), respectively. This study showed that the performance of the “Onsite Leishmania Ab Rapid Test” strip tests was up to the recommended level.

## 1. Introduction

Kala azar or visceral leishmaniasis (VL) is a vector-borne parasitic disease endemic in 45 of 64 districts of Bangladesh where 65 million people have been estimated to be at risk of contracting the disease [1]. The disease presents with prolonged fever, anemia, splenomegaly, anorexia, and wasting and is typically found among the rural poor of the endemic countries. India, Bangladesh, and Nepal together account for about 60% of all the new cases of kala azar which is an estimated 500,000 every year [2]. The governments of India, Bangladesh, and Nepal signed an agreement in 2005 to make joint efforts to eliminate kala azar from this region. The strategy for the elimination programme relies on three main components: rapid diagnosis, effective treatment, and integrated vector control. 

An immunochromatographic test based on a recombinant 39-amino acid repeat antigen, conserved in the kinesin region of *Leishmania chagasi*, known as rK-39 strip test has been the most widely accepted rapid diagnostic test for VL in South Asia [3, 4]. The WHO has recommended rK39 test as rapid diagnostic test (RDT) for VL in South Asia. The Government of Bangladesh (GoB) procured a new RDT kit with the brand name of “Onsite Leishmania Ab Combo” (CTK Biotech Inc., San Diego, Calif, USA) for the kala azar elimination programme through open bidding as per existing policy. The aim of the study was to evaluate the sensitivity and specificity of “Onsite Leishmania Ab Rapid Test” strips to determine whether these kits conformed to the standard set by the kala azar elimination programme.

## 2. Materials and Methods

We used plasma samples of 100 confirmed cases of VL who were randomly selected out of 200 patients enrolled into a clinical trial to evaluate the safety and efficacy of treatment with sodium stibogluconate (SSG) in Bangladesh between May 2007 and May 2009 (ClinicalTrials.gov, Identifier: NCT01240473). All of these patients resided in Mymensingh and presented with a history of fever for two weeks or more and tested positive for VL by parasitological examination of their splenic tissue. We also enrolled 51 healthy controls from the same endemic region of Mymensingh and 50 healthy controls from Mirpur of Dhaka City, a nonendemic area for VL. Healthy volunteers were selected from individuals who did not have a past history of VL. All subjects who participated in the study gave their informed consent before joining the study. The original identification labels of the samples were stripped off before performing the test. The technician who performed the test was completely blind about the cases and the healthy volunteers.

The VL and healthy volunteer samples used in this study were collected from SSG evaluation study approved by the institutional Ethical Review Committee and Research Review Committee of the International Centre for Diarrhoeal Disease Research, Bangladesh (ICDDR,B) where further use of the samples was consented from the participants. 

Blood from VL patients and healthy volunteers was collected in a EDTA-containing tube and then centrifuged for separation of plasma at the field laboratory in Mymensingh where the “*Onsite Leishmania Ab Rapid Test*” strip test was performed according to manufacturer's instruction. Briefly, 5 *μ*L of plasma samples was applied to the base of nitro-cellulose strips impregnated with recombinant leishmanial antigen, then 2-3 drops of the supplied sample diluent buffer (phosphate-buffered saline, plus bovine serum albumin) were added, and the strip was placed flat. The appearance of a lower red band (Control band) indicated the proper functioning of the test while the appearance of an upper red band (Test band) indicated the presence of antileishmanial antibody, signifying a positive test. Usually tests were read within 15 minutes, but positive results could be visible as short as 1 minute.

## 3. Results

Sensitivity and specificity were computed along with 95% confidence interval (CI) using the Epi Info software (version 6.02; CDC, Atlanta, Ga, USA). Data were also analyzed by 2 × 2 contingency tables using the SPSS software (version 10.0) for Windows (release 10.0.1, standard version, 1999; SPSS Inc., Chicago, Ill, USA).

“Onsite Leishmania Ab Rapid Test” strip tests were positive in 94 out of 100 confirmed cases of VL, whereas 4 of 51 healthy subjects from the VL endemic areas were also tested positive. None of the 50 healthy volunteers from the nonendemic areas were positive with “Onsite Leishmania Ab Rapid Test” strip test. Thus, the sensitivity and specificity of  “Onsite Leishmania Ab Rapid Test” strip test were found to be 94% (95% CI: 86.9–97.5) and 96% (95% CI: 89.5–98.7), respectively. Positive predictive value and negative predictive value were found 95.9% (95% CI: 89.3–98.7) and 94.2% (95% CI: 87.2–97.6), respectively, (Table 1).

## 4. Discussion

Elimination of kala azar from Bangladesh and its neighboring countries largely depends on strict adherence to the regional strategic plan that strongly recommends the use of rapid diagnostic tests for diagnosis of VL. The commitment of the GoB to follow the national strategy for elimination of VL is reflected by the present study which was commissioned to ICDDR,B for quality assurance of the diagnostic kits procured by the programme. According to the manufacturer, “Onsite Leishmania Ab Rapid Test” detects antibodies against the same 39 kD protein as reported by the manufacturer of rK39 test (personal communication). Although “Onsite Leishmania Ab Rapid Test” strips have been commercially available worldwide, there is no report in the current literature about its efficacy in diagnosing active cases of VL in South Asia. Multicentre studies on various methods used for diagnosis of VL show wide variation in their sensitivity and specificity depending on geographic location [5]. It is, therefore, of great importance to do a quality check before a new diagnostic kit is used by the national programme. It has been recommended that an ideal VL screening test for case detection should have a sensitivity ≥ 95% and specificity ≥ 98% [6]. Serological test performance often varies with geographical differences. So multicentre studies need to be conducted to check the performance of this “Onsite Leishmania Ab Rapid Test” in different geographic location. We are also curious to see the performance of these new kits for the detection of antileishmanial Ab in urine as urine-based rK39 has already been evaluated recently [7]. This limited study showed that the sensitivity of the “Onsite Leishmania Ab Rapid Test” strips, also referred to as RDT for kala azar, procured by the programme was considered to be up to the recommended level. Samples from other diseases were not included in the assessment of specificity, which is a limitation of this study. This critical information on the quality of “Onsite Leishmania Ab Rapid Test” test strip for diagnosis of kala azar in Bangladesh helped the programme to take informed decision before using the same diagnostic kit in the endemic areas.

## Figures and Tables

**Table 1:** Sensitivity and specificity of “Onsite Leishmania Ab Rapid Test” strips for diagnosis of VL in Bangladesh.

Patients and healthy controls	Onsite Leishmania Ab Rapid Test (RDT)^∗*χ*^		Total
	Positive	Negative	
Kala azar confirmed cases	94	6	100
Healthy control from endemic area	4	47	51
Healthy control from nonendemic area	0	50	50

*Sensitivity = 94% (95% CI: 86.9–97.5); specificity = 96% (95% CI: 89.6–98.8); positive predictive value (PPV) = 95.9% (95% CI: 89.3–98.7); negative predictive value (NPV) = 94.2% (95% CI: 87.2–97.6).

**^*χ*^**Sensitivity, specificity, PPV, NPV, and 95% CI of all were calculated by the Clinical Calculator One (http://faculty.vassar.edu/lowry/clin1.html).
